# Divergent polo box domains underpin the unique kinetoplastid kinetochore

**DOI:** 10.1098/rsob.150206

**Published:** 2016-03-16

**Authors:** Olga O. Nerusheva, Bungo Akiyoshi

**Affiliations:** Department of Biochemistry, University of Oxford, Oxford, UK

**Keywords:** kinetochore, kinetoplastid, *Trypanosoma brucei*, polo-like kinase, polo box domain, DPB

## Abstract

Kinetochores are macromolecular machines that drive eukaryotic chromosome segregation by interacting with centromeric DNA and spindle microtubules. While most eukaryotes possess conventional kinetochore proteins, evolutionarily distant kinetoplastid species have unconventional kinetochore proteins, composed of at least 19 proteins (KKT1–19). Polo-like kinase (PLK) is not a structural kinetochore component in either system. Here, we report the identification of an additional kinetochore protein, KKT20, in *Trypanosoma brucei*. KKT20 has sequence similarity with KKT2 and KKT3 in the Cys-rich region, and all three proteins have weak but significant similarity to the polo box domain (PBD) of PLK. These divergent PBDs of KKT2 and KKT20 are sufficient for kinetochore localization *in vivo*. We propose that the ancestral PLK acquired a Cys-rich region and then underwent gene duplication events to give rise to three structural kinetochore proteins in kinetoplastids.

## 1. Introduction

Eukaryotic chromosome segregation is directed by the kinetochore, the macromolecular protein complex that assembles onto centromeric DNA and captures spindle microtubules during mitosis and meiosis [1,2]. The kinetochore is a highly complicated structure that consists of more than 30 different structural proteins even in a simple budding yeast kinetochore [3]. It is thought that most eukaryotes use these proteins to build kinetochores because of their conservation in diverse eukaryotes [4–7]. However, none of these conventional kinetochore components has been found in kinetoplastids, a group of evolutionarily distant eukaryotes that include medically important pathogens such as *Trypanosoma brucei* and *Leishmania* [8]. These organisms instead have unconventional kinetochores, composed of at least 19 proteins named KKT1–19 (kinetoplastid kinetochore protein) [7]. Their sequence analyses failed to identify orthologous proteins in other organisms, suggesting that kinetoplastid kinetochores may have a distinct evolutionary origin [7]. The evolutionary origins of KKT proteins are not known.

Polo-like kinases (PLKs) are Ser/Thr kinases that regulate cell cycle progression, centriole biogenesis, kinetochore functions, cytokinesis, the DNA damage response and neuronal activity [9–12]. PLKs consist of an N-terminal kinase domain and C-terminal polo box domain (PBD) [13]. PLKs carry out diverse functions by phosphorylating numerous substrates, where specificity comes in part from the PBD that governs the localization of PLKs in space and time. The PBDs are protein–protein interaction domains that require priming phosphorylation [14], although phosphorylation-independent interactions are also possible [15]. PLK1 is known to localize at the kinetochore, interact with kinetochore/checkpoint proteins and regulate kinetochore functions in some eukaryotes [16–19]. However, it is not a structural kinetochore component in any organism studied thus far, and there is no report of a kinetochore protein that contains a PBD [20].

Here we report the identification of a previously unidentified kinetochore protein in *T. brucei* and discuss how it defines a family of three kinetoplastid kinetochore proteins that might have evolved from PLK.

## 2. Results

### 2.1. Identification of KKT20 in *Trypanosoma brucei*

**Figure 1.**
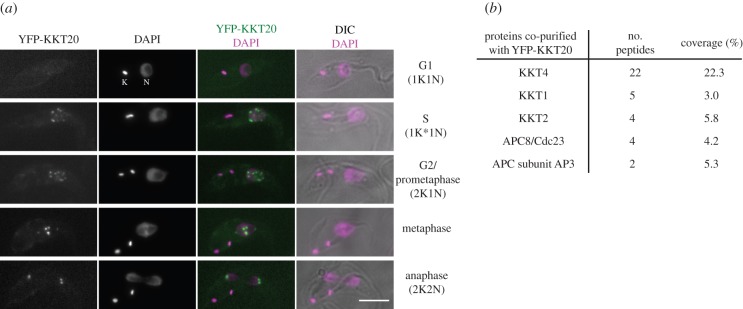
Identification of KKT20. (*a*) YFP-KKT20 localizes at kinetochores from S phase until the end of anaphase. Examples of trypanosome procyclic cells expressing YFP-KKT20 are shown. K and N stand for the kinetoplast and nucleus, respectively. Note that there are weak spindle-like signals near the poles besides kinetochore dots during metaphase. K* denotes an elongated kinetoplast. Scale bar, 5 µm. (*b*) YFP-KKT20 MS summary table. See electronic supplementary material, table S1 for all proteins identified by MS.

To understand the design principle of kinetoplastid kinetochores, it is essential to obtain a complete list of kinetochore components. We previously performed immunoprecipitation of the YFP-tagged version of KKT proteins in *T. brucei* and identified co-purifying proteins by mass spectrometry. Subsequent YFP tagging of candidate proteins led to the identification of 19 kinetochore proteins, named KKT1–19 [7]*.* KKT4 was unique among the 19 proteins, because KKT4, not other KKT proteins, co-purified with significant amounts of APC/C subunit proteins. Because the complete list of APC/C components in *T. brucei* remained unclear until recently [21], we thought that those proteins that were uniquely identified in the KKT4 immunoprecipitation sample may well be as-yet unidentified APC/C components. However, one such protein (ORF Tb927.8.4760) had typical kinetochore localization *in vivo* (figure 1*a*). We named this protein KKT20. Unlike KKT4, which localized at the kinetochore throughout the cell cycle, KKT20 localized from S phase until the end of anaphase (figure 1*a*). Mass spectrometry of proteins that co-purified with YFP-KKT20 confirmed its interaction with several KKT proteins, among which KKT4 was the most significant hit (figure 1*b*). In addition, some APC/C subunits were detected. Together with our previous result that KKT20 and APC/C subunits were detected only in the KKT4 sample, it is likely that KKT20 and KKT4 are in close proximity at the kinetochore. Supporting this possibility, KKT20 had weak spindle-like signal near the poles during metaphase (figure 1*a*), which was observed for KKT4 but not any other KKT protein [7].

### 2.2. KKT20 has similarity to KKT2 and KKT3

**Figure 2.**
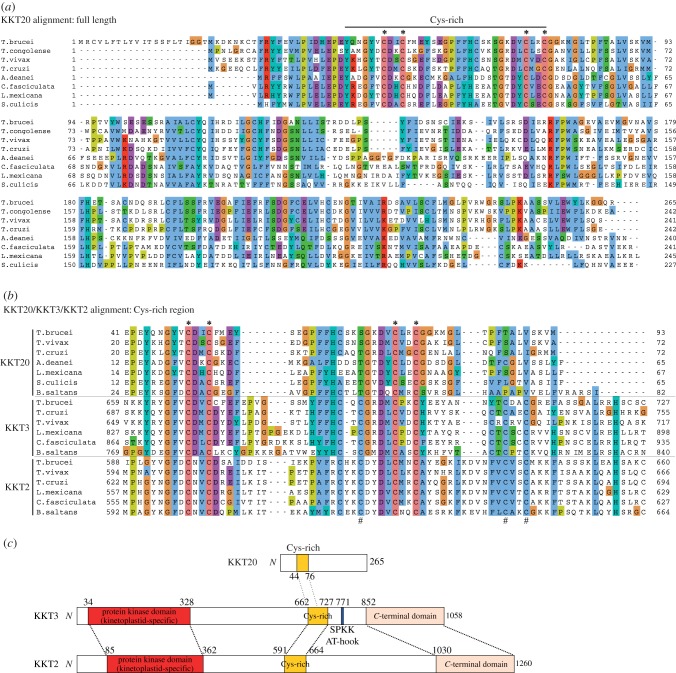
KKT20 has similarity to KKT2 and KKT3 in the Cys-rich region. (*a*) Multiple alignment of KKT20 reveals four Cys residues conserved among kinetoplastids (indicated by asterisk). (*b*) Multiple alignment of KKT20/KKT3/KKT2 in the Cys-rich region shows the conservation of some Cys residues in all three proteins (denoted by asterisk), as well as several additional Cys conserved in KKT2 and KKT3, not KKT20 (denoted by hash). (*c*) Schematic of *T. brucei* KKT20, KKT3 and KKT2 proteins, showing similarity in the Cys-rich region. KKT2 and KKT3 additionally share similarity in the N-terminal kinase domain and C-terminal domain.

KKT20 is conserved among kinetoplastids and has four conserved Cys residues (figure 2*a*). Cys-rich motifs are present in hundreds of proteins in eukaryotic genomes [22], including two homologous KKT proteins, KKT2 and KKT3, in kinetoplastids [7]. Interestingly, PSI-BLAST search using *T. brucei* KKT20 on non-redundant protein sequence database containing proteome from numerous organisms collected KKT20 homologues in the first iteration and then identified the KKT3 proteins from *Trypanosoma rangeli* and *Trypanosoma grayi* in the second iteration, revealing similarity in the Cys-rich region. A similar result was obtained for *T. cruzi* KKT20, suggesting that the KKT20's Cys-rich region apparently has a higher level of similarity to KKT3's Cys-rich region than to other proteins. Alignment of KKT20/KKT3/KKT2 revealed that KKT20's four conserved Cys residues are also present in KKT3 and KKT2 (figure 2*b*, indicated by asterisk). In addition to these four Cys, KKT3 and KKT2 have several additional conserved Cys residues (figure 2*b*, indicated by hash). These results revealed that KKT20 has similarity to KKT3 and KKT2 at least in the Cys-rich region (figure 2*c*).

### 2.3. The C-terminal domain of KKT20, KKT2 and KKT3 has similarity to the polo box domain

**Figure 3.**
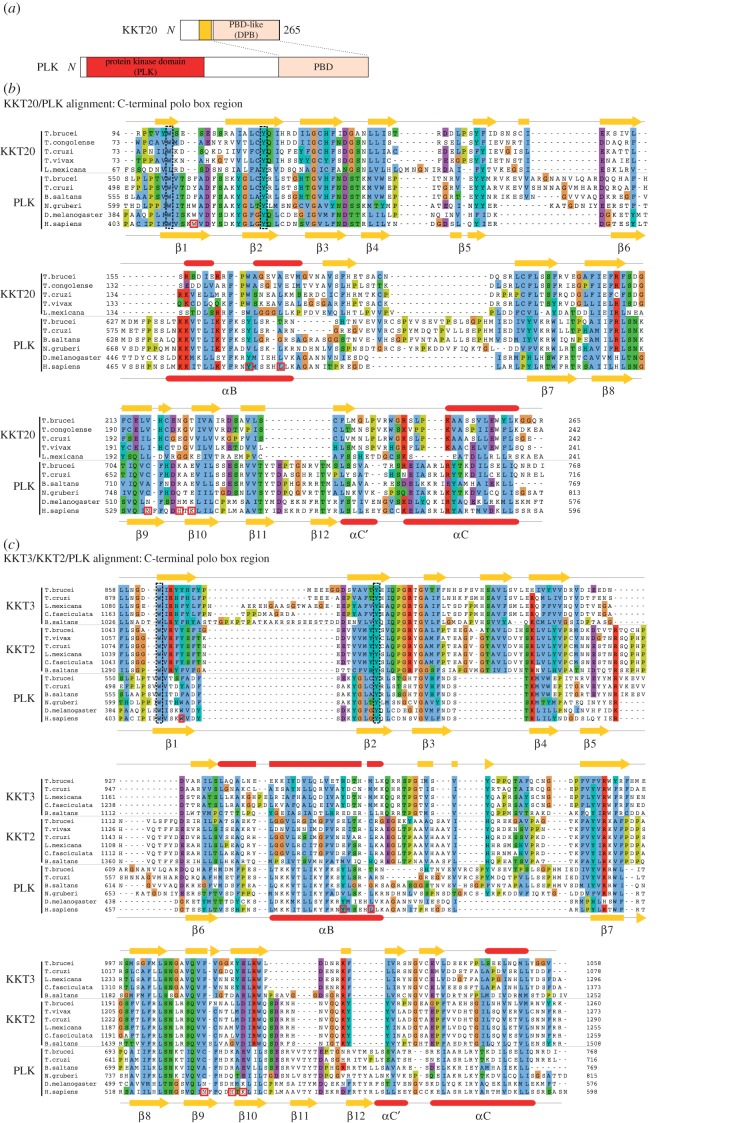
The C-terminal domain of KKT20/KKT2/KKT3 has similarity to the PBD. (*a*) Schematic of KKT20 and PLK, showing similarity in the C-terminal region. (*b*) Multiple alignment of the C-terminal domain of KKT20 and PLK PBDs with their secondary structure predictions reveals significant similarity between them. *T. brucei* KKT20 (top) and *H. sapiens* PLK1 (bottom) structural predictions were derived from PSIPRED. The fold nomenclature of *H. sapiens* PLK1 is based on [14], and the residues critical for phosphopeptide binding are shown in red boxes. (*c*) Multiple alignment of the C-terminal domain of KKT3/KKT2 and PLK PBDs with secondary structure predictions for *T. brucei* KKT3 (top) and *H. sapiens* PLK1 (bottom). Highly conserved residues that were mutated in this study are highlighted by black dotted boxes.

We then performed sensitive profile sequence searches using hidden Markov models [23,24]. To our surprise, a Jackhmmer search using *T. brucei* KKT20 identified the PLK from *Candida tenuis* (Fungi) in the second iteration (*E*-value < 10^−2^). A similar search using *Trypanosoma congolense* KKT20 hit the PLK from *Trichoplax adhaerens* (Metazoa). KKT20 does not have a kinase domain and the similarity was found between KKT20's C-terminal region and the polo boxes of these PLKs (figure 3*a*). Similar results were obtained using HHpred that allows profile-to-profile comparison [25], where the highest level of similarity was found for *T. congolense* KKT20 against polo boxes (pfam PF00659: probability 0.96, *E*-value < 10^−2^). Furthermore, secondary structure predictions revealed similarity between KKT20's C-terminal region and polo boxes (figure 3*b*). These results showed that the C-terminal region of KKT20 has weak but significant similarity to PBD.

KKT2 and KKT3 have three domains conserved among kinetoplastids: a protein kinase domain, a central domain containing Cys-rich region and a C-terminal domain (figure 2*c*) [7]. Although KKT20 has similarity to KKT2/KKT3 in the Cys-rich region, we could not detect significant similarity elsewhere using PSI-BLAST or HMMER (figure 2*c*). However, a Jackhmmer search using the KKT2 C-terminal domain from *Bodo saltans* collected KKT2 homologues in the first iteration, KKT3 homologues in the second, and then detected *Leishmania* PLKs in the third iteration (*E*-value < 10^−2^). Furthermore, similar searches using the C-terminal domains of KKT2 from other kinetoplastids or KKT3 consistently revealed marginal (but non-significant) sequence similarity to PLKs from various eukaryotes. None of these searches hit KKT20, consistent with the lack of significant sequence similarity between KKT20 and KKT2/KKT3 except in the Cys-rich region. Again, structural predictions revealed similar secondary structures between KKT2/KKT3's C-terminal domain and polo boxes (figure 3*c*). Therefore, like KKT20, KKT2/KKT3 also have a domain that has weak similarity to polo boxes. We propose to call the C-terminal domain of KKT20/KKT2/KKT3 the divergent polo box (DPB) domain.

### 2.4. Isolated divergent polo box domains of KKT2 and KKT20 are sufficient for kinetochore localization

**Figure 4.**
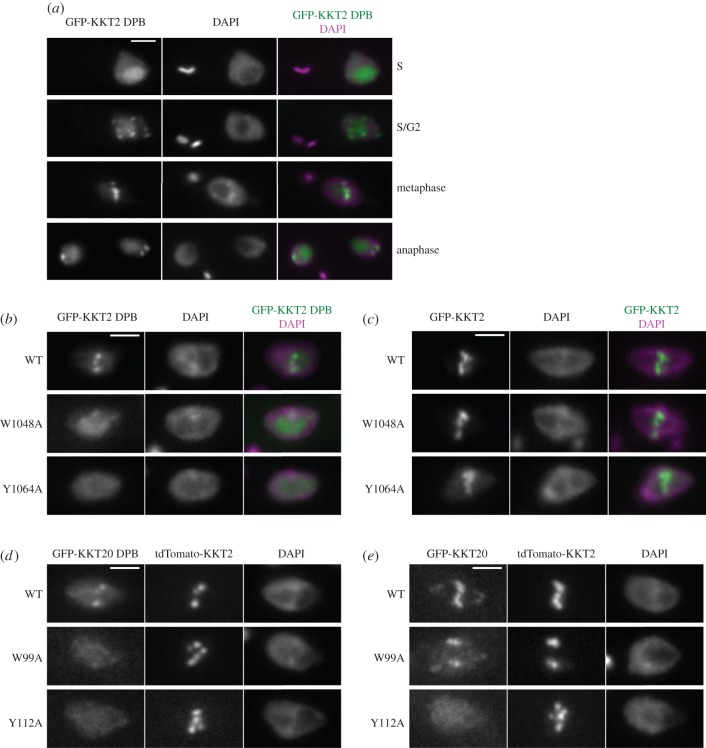
Isolated DPB domains of KKT2 and KKT20 can localize at kinetochores. (*a*) KKT2 DPB has kinetochore localization in G2, metaphase and anaphase. (*b*,*c*) W1048A and Y1064A mutations abolish the kinetochore localization of KKT2 DPB (*b*) but not the full-length protein (*c*). (*d*) KKT20 DPB localizes at kinetochores, which is abolished in W99A and Y112A mutants. Note that KKT20 DPB co-localizes with tdTomato-KKT2. (*e*) The Y112A mutation, but not W99A, abolishes the kinetochore localization of the full-length KKT20 protein. For (*a*–*e*), inducible GFP–NLS fusion proteins were expressed with 10 ng ml^−1^ doxycycline for 1 day. Scale bars, 2 µm.

To investigate the role of DPB domains, we ectopically expressed the GFP–NLS fusion proteins in trypanosomes. We found that KKT2 DPB (residues 1024–1260) had typical kinetochore localization during G2 and mitosis (figure 4*a*), showing that the isolated DPB domain of KKT2 is sufficient for mediating kinetochore localization, presumably through interaction with other kinetochore proteins. We then mutated some residues conserved in both DPB domains and PBDs (highlighted by black dotted boxes in figure 3*c*). The resulting KKT2 DPB^W1048A^ and KKT2 DPB^Y1064A^ mutants did not localize at kinetochores (figure 4*b*), suggesting that these conserved residues are important for its function.

In contrast to the DPB domain of KKT2, which localizes at kinetochores starting from G2 phase, full-length KKT2 localizes throughout the cell cycle [7] (data not shown). Furthermore, mutating the conserved residues in DPB did not abolish the kinetochore localization of the full-length protein (KKT2^W1048A^ and KKT2^Y1064A^; figure 4*c*). These results suggest that KKT2 possesses other region(s) that promote its kinetochore localization, which is in line with the presence of putative DNA-binding motifs in the central region [7].

We did not observe any kinetochore localization for the DPB of KKT3 (residues 831–1058; data not shown). The KKT20 DPB (residues 83–265) had marginal kinetochore localization, which was abolished when highly conserved residues were mutated (KKT20-DPB^W99A^ and KKT20-DPB^Y112A^; figure 4*d*). The Y112A mutation (not W99A) also abolished the localization of the full-length protein (figure 4*e*), suggesting that the DPB plays a major role in promoting the kinetochore localization of KKT20. Taken together, these data provide functional evidence for the DPB domains of KKT2 and KKT20.

## 3. Discussion

This study revealed that three kinetoplastid kinetochore proteins (KKT2, KKT3 and KKT20) have a divergent PBD that is distantly related to the PBDs of PLKs. Bona fide PLK is present in kinetoplastids [26,27]. In *T. brucei,* PLK is known to play critical functions for basal body biogenesis and cytokinesis but is not known to localize at the kinetochore or regulate kinetochore functions [28–30]. The PBDs in kinetoplastid PLKs do not have residues that are crucial for phosphopeptide binding in human PLK1 [14], and it remains unknown whether they interact with other proteins in a phospho-dependent manner [31,32]. The DPBs of KKT20/KKT2/KKT3 also lack these residues (figure 3*b*,*c*). In the future, it will be important to identify their interaction partners (if any) and reveal the mechanism of interaction to shed light into the function of these proteins.

Besides the DPB domains, KKT20 and KKT2/KKT3 also have similarity in the Cys-rich region, suggesting that these proteins may share common ancestry. KKT2 and KKT3 additionally have an N-terminal kinase domain that does not have clear affiliation to any known kinase group [33]. Given the notion that PLK was present in the last eukaryotic common ancestor [34], we speculate the following scenario as a possible evolutionary origin of KKT2/KKT3/KKT20: PLK (or an ancestor of PLK) that had an N-terminal kinase domain and C-terminal polo boxes acquired a Cys-rich region in the middle. This ancestor highly diverged in amino acid sequences, so that the kinase domain looks unique among eukaryotic kinases, and underwent gene duplication to give rise to KKT2 and KKT3. These proteins in some kinetoplastids additionally acquired DNA-binding motifs such as AT-hook and SPKK, possibly to enhance their interaction with centromeric DNA [7]. While all three proteins retained the Cys-rich region and the DPB domain, KKT20 lost a kinase domain at some point. Now, these three proteins appear to perform distinct functions, judging from their distinct interaction profiles and localization patterns [7].

Gene duplication is thought to be a major mechanism to generate new functions using pre-existing proteins [35]. In non-kinetoplastid eukaryotes, the following structural modules are present in kinetochore proteins and spindle checkpoint proteins: the RWD domain in Spc24/Spc25, Ctf19/Mcm21, Csm1, Mad1 and KNL1 [36–39], CH domain in Ndc80/Nuf2 [40–42], TPR domain in Bub1/BubR1 and Mps1 [43,44], and histone-fold domain in CENT-T/W/S/X and CENP-A [45–47]. This suggests that the highly complicated present-day kinetochores originated from a small number of protein modules aided by gene duplication. So far, we could not detect any of these domains in the 20 kinetoplastid kinetochore proteins, consistent with the possibility that the kinetoplastid kinetochores have a distinct evolutionary origin [7]. The presence of kinetochore proteins that have DPB domains in kinetoplastids, but not in other eukaryotes, further supports this possibility. Nonetheless, gene duplication products are found in kinetoplastid kinetochores, namely KKT17/KKT18, KKT10/KKT19 and KKT2/KKT3/KKT20, highlighting the importance of gene duplication in the invention of macromolecular complexes.

## 4. Material and methods

### 4.1. Cells

All cell lines used in this study were derived from *T. brucei* SmOxP927 procyclic form cells (TREU 927/4 expressing T7 RNA polymerase and the tetracycline repressor to allow inducible expression) [48]. Cells were grown at 28°C in SDM-79 medium supplemented with 10% (v/v) heat-inactivated fetal calf serum [49]. Endogenous YFP tagging was performed using the pEnT5-Y vector [50], with the following primers: BA887 and BA888 for 247 bp starting at the second codon of KKT20 coding sequence, and BA889 and BA890 for 250 bp of 5′ UTR. These two DNA fragments amplified from genomic DNA were ligated into pEnT5-Y cut with *Xba*I and *Bam*HI restriction sites, making pBA463. Endogenous tdTomato tagging of KKT2 was performed by subcloning the KKT2 targeting sequence of pBA67 into pBA148 using *Xba*I and *Bam*HI restriction sites, making pBA164 [7].

To make pBA310 (inducible expression vector with GFP–NLS), a synthetic DNA fragment that has an NLS sequence from La protein (RGHKRSRE) [51] and multiple cloning sites (made by annealing BA680 and BA681) was ligated into pDEX777 cut with *Xba*I and *Bam*HI. Full-length KKT2 (pBA425: amplified from genomic DNA with BA763 and BA768), KKT2 DPB^1024–1260^ (pBA736: BA1159 and BA768) and KKT3 DPB^831–1058^ (pBA366: BA619 and BA620) were ligated into pBA310 cut with *Bam*HI and *Afl*II, whereas full-length KKT20 (pBA747: BA985 and BA988) and KKT20 DPB^83–265^ (pBA748: BA1157 and BA988) were ligated into pBA310 cut with *Pac*I and *Asc*I. Site-directed mutagenesis was performed using Phusion polymerase and the following primers: KKT2^W1048A^ (BA1292 and BA1293), KKT2^Y1064A^ (BA1294 and BA1295), KKT20^W99A^ (BA1296 and BA1297) and KKT20^Y112A^ (BA1298 and BA1299). All constructs were sequence verified.

Plasmids linearized by *Not*I were transfected to trypanosomes by electroporation into an endogenous locus (pBA463 and pBA164) or 177 bp repeats on minichromosomes (pBA310 derivatives). Transfected cells were selected by the addition of 25 µg ml^−1^ hygromycin (pBA463), 10 µg ml^−1^ blasticidin (pBA164) or 5 µg ml^−1^ phleomycin (pBA310 derivatives). The expression of GFP–NLS fusion proteins (pBA310 derivatives) was induced by the addition of doxycycline (10 ng ml^−1^) for 1 day. All cell lines, plasmids and primers used in this study are listed in electronic supplementary material, tables S3, S4 and S5, respectively.

### 4.2. Fluorescence microscopy

For the analysis of fluorescently tagged proteins, cells were washed once with PBS, settled onto glass slides and fixed with 4% paraformaldehyde in PBS for 5 min. Cells were then permeabilized with 0.1% NP-40 in PBS for 5 min and embedded in mounting media (1% w/v 1,4-diazabicyclo[2.2.2]octane, 90% glycerol, 50 mM sodium phosphate pH 8.0) containing 100 ng ml^−1^ 4,6-diamidino-2-phenylindole dihydrochloride (DAPI). Images were captured on a DeltaVision fluorescence microscope (Applied Precision) installed with softWoRx v. 5.5 housed in the Oxford Micron facility. Fluorescent images were captured with a CoolSNAP HQ camera and processed in ImageJ [52]. Cell cycle stages of individual cells were estimated as described previously [53,54].

### 4.3. Immunoprecipitation and mass spectrometry

Immunoprecipitation and mass spectrometry were performed essentially as described previously [7] except that a Q-Exactive (Thermo Scientific) at the Central Proteomics Facility (www.proteomics.ox.ac.uk, University of Oxford) was used and that peptides were identified with Mascot (Matrix Science). Proteins identified with at least two peptides were considered and shown in electronic supplementary material, table S1. Raw MS data are available upon request.

### 4.4. Bioinformatics

PSI-BLAST search was done on non-redundant protein sequences database (all non-redundant GenBank CDS translations + PDB + SwissProt + PIR + PRF excluding environmental samples from WGS projects, 14 July 2015), using default setting [55]. Jackhmmer search (v. 3.0) was done on UniProt reference proteomes, using default setting (*E*-value cut-off 0.01) [24]. HHpred was carried out using pfamA_28.0 HMM database [25]. Multiple sequence alignment was performed with MAFFT (L-INS-i method, v. 7) [56] and visualized with ClustalX colouring scheme in Jalview (v. 2.8) [57]. Secondary structure predictions were performed using PSIPRED (v. 3.3) [58]. Accession numbers for protein sequences retrieved from TriTryp database [59–61], GeneDB [62,63], NCBI database [64] or GenBank [65] are listed in electronic supplementary material, table S2.

## Supplementary Material

Nerusheva_tableS1.xlsx

## Supplementary Material

Nerusheva_table_S2_S3_S4_S5.pdf

## Supplementary information

A supplementary Excel file has the list of YFP-KKT20 MS/MS hits (electronic supplementary material, table S1).

A supplementary PDF file has accession numbers for protein sequences (electronic supplementary material, table S2), cell lines (electronic supplementary material, table S3), plasmids (electronic supplementary material, table S4) and primer sequences (electronic supplementary material, table S5) used in this study.
