# Supplementary material for: Divergent polo box domains underpin the unique kinetoplastid kinetochore
Source: Open Biol. 2016 Mar 16;6(3):150206. doi: 10.1098/rsob.150206 (PMC4821238; doi:10.1098/rsob.150206)
Supplement: Nerusheva_table_S2_S3_S4_S5.pdf [file rsob150206supp2.pdf]

Table S2. Accession numbers for protein sequences retrieved from TriTryp database [1–3], GeneDB [4,5], NCBI database [6] or GenBank [7]

Gene	Organism	Accession number
KKT20	*Trypanosoma brucei*	Tb927.8.4760
	Trypanosoma congolense	TcIL3000_0_00100
	Trypanosoma vivax	TvY486_0804180
	Trypanosoma cruzi	TcCLB.419043.10
	Angomonas deanei	EPY28808
	Crithidia fasciculata	CFAC1_040021300
	Leishmania mexicana	LmxM.10.1227
	Strigomonas culicis	EPY28494
	Bodo saltans	BS19285
KKT3	*Trypanosoma rangeli*	ESL05960
	Trypanosoma grayi	XP_009309824
	Crithidia fasciculata	CFAC1_300091200
KKT2	*Crithidia fasciculata*	CFAC1_280060200
PLK	*Candida tenuis*	XP_006685766
	Trichoplax adhaerens	XP_002116022
	Trypanosoma brucei	Tb927.7.6310
	Trypanosoma cruzi	TcCLB.506513.160
	Bodo saltans	BS35140
	Naegleria gruberi	XP_002670439
	Drosophila melanogaster (Polo)	NP_524179
	Homo sapiens (Plk1)	NP_005021

KKT3 and KKT2 from other species were described previously [8].

Table S3. Trypanosome cell lines used in this study.

Strain	Description	Used in Figure
SmOxP9	Parental cell line that expresses TetR and T7 RNAP [9]	–
BAP391	TY-YFP-KKT20	1
BAP517	GFP-NLS-KKT2 DPB	4a, 4b
BAP296	GFP-NLS-KKT3 DPB	data not shown
BAP535	GFP-NLS-KKT2 DPB W1048A	4b
BAP538	GFP-NLS-KKT2 DPB Y1064A	4b
BAP327	GFP-NLS-KKT2	4c
BAP533	GFP-NLS-KKT2 W1048A	4c
BAP536	GFP-NLS-KKT2 Y1064A	4c
BAP308	TY-tdTomato-KKT2	–
BAP546	TY-tdTomato-KKT2, GFP-NLS-KKT20 DPB	4d
BAP550	TY-tdTomato-KKT2, GFP-NLS-KKT20 DPB W99A	4d
BAP551	TY-tdTomato-KKT2, GFP-NLS-KKT20 DPB Y112A	4d
BAP545	TY-tdTomato-KKT2, GFP-NLS-KKT20	4e
BAP547	TY-tdTomato-KKT2, GFP-NLS-KKT20 W99A	4e
BAP549	TY-tdTomato-KKT2, GFP-NLS-KKT20 Y112A	4e

Table S4. Plasmids used in this study.

Plasmid	Description
pEnT5-Y	TY-YFP tagging vector, Hygromycin [9]
pBA67	TY-YFP-KKT2 tagging vector, Hygromycin [8]
pBA148	TY-tdTomato tagging vector, Blasticidin [8]
pBA164	TY-tdTomato-KKT2 tagging vector, Blasticidin
pBA463	TY-YFP-KKT20 tagging at endogenous locus, Hygromycin
pDEX777	Inducible expression vector (GFP-TY fusion), integrate at 177 bp, Phleomycin [9]
pBA310	Inducible expression vector (GFP-NLS fusion), integrate at 177 bp, Phleomycin
pBA736	Inducible GFP-NLS-KKT2 DPB, integrate at 177 bp, Phleomycin
pBA366	Inducible GFP-NLS-KKT3 DPB, integrate at 177 bp, Phleomycin
pBA782	Inducible GFP-NLS-KKT2 DPB W1048A, integrate at 177 bp, Phleomycin
pBA785	Inducible GFP-NLS-KKT2 DPB Y1064A, integrate at 177 bp, Phleomycin
pBA425	Inducible GFP-NLS-KKT2, integrate at 177 bp, Phleomycin
pBA780	Inducible GFP-NLS-KKT2 W1048A, integrate at 177 bp, Phleomycin
pBA783	Inducible GFP-NLS-KKT2 Y1064A, integrate at 177 bp, Phleomycin
pBA748	Inducible GFP-NLS-KKT20 DPB, integrate at 177 bp, Phleomycin
pBA791	Inducible GFP-NLS-KKT20 DPB W99A, integrate at 177 bp, Phleomycin
pBA792	Inducible GFP-NLS-KKT20 DPB Y112A, integrate at 177 bp, Phleomycin
pBA747	Inducible GFP-NLS-KKT20, integrate at 177 bp, Phleomycin
pBA786	Inducible GFP-NLS-KKT20 W99A, integrate at 177 bp, Phleomycin
pBA790	Inducible GFP-NLS-KKT20 Y112A, integrate at 177 bp, Phleomycin

Table S5. Primers used in this study.

To make	Primers (all are listed 5' to 3')
pBA463	CDS targeting sequence with XbaI and NotI<br>BA887: GATCGATC TCTAGA GGAGCAGGT AGGTGCGTATTATTTACACT<br>BA888: GATCGATC GCGGCCGC GAGTAAGCCCATTTTACCG<br>5'UTR targeting sequence with NotI and BamHI<br>BA889: GATCGATC GCGGCCGC CTGTTTACCGCGCGTCTGAC<br>BA890: GATCGATC GGATCC TCAGTGGTTAGTGGTGGCAG
pBA310	NLS with multiple cloning sites with synthetic XbaI and BamHI sites<br>BA680: CTAGA ATCGATAC CGGT CGAGGACACAAGCGGTCACGTGAA CAATTGCCGCGGGATATCCATATGTTAATTAAGGCGCGCCTGATCAGTTT AAAC G<br>BA681: GATCC GTTTAAACTGATCAGGCGCGCCTTAATTAACATATGGATATCCCGCGGC A ATTGTTCACGTGACCGCTTGTGTCCTCGACCGGTATCGAT T
pBA425	CDS targeting sequence with BamHI and AflII<br>BA763: AGTCAC GGATCC TTCAATGTCTCACCAGCGAG<br>BA768: AGCCAC CTTAAG TCATTTCCGGTAGACGTTGC
pBA736	CDS targeting sequence with BamHI and AflII<br>BA1159: AGTCAC GGATCC ACGGCGTACCTGGCCCTTCC<br>BA768: AGCCAC CTTAAG TCATTTCCGGTAGACGTTGC
pBA366	CDS targeting sequence with BamHI and AflII<br>BA619: AGTCAC GGATCC CTAAGTGGTGAATGGAAACC<br>BA620: AGCCAC CTTAAG TTACACGCCACCGTAGAGCAT
pBA747	CDS targeting sequence with PacI and AscI<br>BA985: GATC TTAATTAA G AGGTGCGTATTATTTACACT<br>BA988: GATC GGCGCGCC CTACCTTTGTCCTCCTTTTAAAT
pBA748	CDS targeting sequence with PacI and AscI<br>BA1157: AGTCAC TTAATTAA G ACTCCTTTCACTGCACTGGT<br>BA988: GATC GGCGCGCC CTACCTTTGTCCTCCTTTTAAAT
KKT2 W1048A	BA1292: GATGACTTCCTAAGCGGGGCG GCG GTAAGGGTTTACTCTTTCATTG<br>BA1293: CAATGAAAGAGTAAACCCTTACCGCCGCCCCGCTTAGGAAGTCATC
KKT2 Y1064A	BA1294: CTGAAGTTGTTGTGATGTAT GCT TCTGTGCAACCCGGTAGGTAC<br>BA1295: GTACCTACCGGGTTGCACAGAAGCATACATCACAACAACTTCAG
KKT20 W99A	BA1296: GTAATGAGGCCTACTGTCTAC GCG AGTGAGAGTGAGAGCTCTAG

	BA1297: CTAGAGCTCTCACTCTCACTCGCGTAGACAGTAGGCCTCATTAC
KKT20 Y112A	BA1298: CTAGAGCAATTGCACTTTGC GCC CAGATCCATCGAGACATTC<br>BA1299: GAATGTCTCGATGGATCTGGGCGCAAAGTGCAATTGCTCTAG

Supplementary Reference

1. Berriman, M. et al. 2005 The genome of the African trypanosome Trypanosoma brucei. *Science* **309**, 416–422. (doi:10.1126/science.1112642)

2. El-Sayed, N. M. et al. 2005 The genome sequence of Trypanosoma cruzi, etiologic agent of Chagas disease. *Science* **309**, 409–415. (doi:10.1126/science.1112631)

3. Ivens, A. C. et al. 2005 The genome of the kinetoplastid parasite, Leishmania major. *Science* **309**, 436–442. (doi:10.1126/science.1112680)

4. Logan-Klumpler, F. J. et al. 2012 GeneDB—an annotation database for pathogens. *Nucl. Acids Res.* **40**, D98–D108. (doi:10.1093/nar/gkr1032)

5. Jackson, A. P. et al. 2015 Kinetoplastid Phylogenomics Reveals the Evolutionary Innovations Associated with the Origins of Parasitism. *Curr. Biol.* (doi:10.1016/j.cub.2015.11.055)

6. Pruitt, K. D., Tatusova, T. & Maglott, D. R. 2005 NCBI Reference Sequence (RefSeq): a curated non-redundant sequence database of genomes, transcripts and proteins. *Nucl. Acids Res.* **33**, D501–D504. (doi:10.1093/nar/gki025)

7. Benson, D. A., Karsch-Mizrachi, I., Lipman, D. J., Ostell, J. & Wheeler, D. L. 2005 GenBank. *Nucl. Acids Res.* **33**, D34–D38. (doi:10.1093/nar/gki063)

8. Akiyoshi, B. & Gull, K. 2014 Discovery of unconventional kinetochores in kinetoplastids. *Cell* **156**, 1247–1258. (doi:10.1016/j.cell.2014.01.049)

9. Poon, S. K., Peacock, L., Gibson, W., Gull, K. & Kelly, S. 2012 A modular and optimized single marker system for generating Trypanosoma brucei cell lines expressing T7 RNA polymerase and the tetracycline repressor. *Open Biol* **2**, 110037. (doi:10.1098/rsob.110037)
